# Prevalence of chronic postsurgical hypoparathyroidism not adequately controlled: an analysis of a nationwide cohort of 337 patients

**DOI:** 10.3389/fendo.2024.1464515

**Published:** 2024-09-25

**Authors:** Juan J. Díez, Emma Anda, Begoña Pérez-Corral, Miguel Paja, Victoria Alcazar, Cecilia Sánchez-Ragnarsson, Aida Orois, Ana R. Romero-Lluch, Marcel Sambo, Amelia Oleaga, Águeda Caballero, María R. Alhambra, Virginia Urquijo, Ana M. Delgado-Lucio, José C. Fernández-García, Viyey K. Doulatram-Gamgaram, Suset Dueñas-Disotuar, Tomás Martín, Mercedes Peinado, Julia Sastre

**Affiliations:** ^1^ Department of Endocrinology, Hospital Universitario Puerta de Hierro Majadahonda, Instituto de Investigación Sanitaria Puerta de Hierro Segovia de Arana, Majadahonda, Spain; ^2^ Department of Medicine, Universidad Autónoma de Madrid, Madrid, Spain; ^3^ Department of Endocrinology, Hospital Universitario de Navarra, Pamplona, Spain; ^4^ Department of Endocrinology, Complejo Asistencial Universitario de León, León, Spain; ^5^ Department of Endocrinology, Hospital Universitario de Basurto, Universidad del País Vasco, Universidad del País Vasco (UPV)/EHU, Bilbao, Spain; ^6^ Department of Endocrinology, Hospital Severo Ochoa, Leganés, Spain; ^7^ Department of Endocrinology, Hospital Universitario Central de Asturias, Instituto de Investigación Sanitaria del Principado de Asturias, Oviedo, Spain; ^8^ Department of Endocrinology and Nutrition, Hospital Clínic, Barcelona, Spain; ^9^ Department of Endocrinology, Hospital Universitario Virgen del Rocío, Sevilla, Spain; ^10^ Department of Endocrinology, Hospital General Universitario Gregorio Marañón, Madrid, Spain; ^11^ Department of Endocrinology, Hospital Universitario de Canarias, Tenerife, Spain; ^12^ Department of Endocrinology, Hospital Universitario Reina Sofía, Córdoba, Spain; ^13^ Department of Endocrinology, Hospital Universitario de Cruces, Bilbao, Spain; ^14^ Department of Endocrinology, Hospital Universitario de Burgos, Burgos, Spain; ^15^ Department of Endocrinology, Hospital Regional Universitario de Málaga, Instituto de Investigación Biomédica de Málaga, Universidad de Málaga, Málaga, Spain; ^16^ Department of Endocrinology, Hospital Universitario Virgen Macarena, Sevilla, Spain; ^17^ Department of Endocrinology, Hospital Universitario de Toledo, Toledo, Spain

**Keywords:** postsurgical hypoparathyroidism, adequacy, comorbidity, disease control, patient well-being

## Abstract

**Purpose:**

The identification of patients with chronic hypoparathyroidism who are adequately (AC) or not adequately controlled (NAC) has clinical interest, since poor disease control is related to complications and mortality. We aimed to assess the prevalence of NAC patients in a cohort of subjects with postsurgical hypoparathyroidism.

**Methods:**

We performed a multicenter, retrospective, cohort study including patients from 16 Spanish hospitals with chronic hypoparathyroidism lasting ≥3 years. We analyzed disease control including biochemical profile and clinical wellness. For biochemical assessment we considered three criteria: criterion 1, normal serum calcium, phosphorus and calcium x phosphorus product; criterion 2, the above plus estimated glomerular filtration rate ≥60 ml/min/1.73 m^2^; and criterion 3, the above plus normal 24-hour urinary calcium excretion. A patient was considered AC if he or she met the biochemical criteria and was clinically well.

**Results:**

We included 337 patients with postsurgical hypoparathyroidism (84.3% women, median age 45[36-56] years, median time of follow-up 8.9[6.0-13.0] years). The proportions of NAC patients with criteria 1, 2 and 3 were, respectively, 45.9%, 49.2% and 63.1%. Patients who had dyslipidemia at the time of diagnosis presented a significantly higher risk of NAC disease (criterion 3; OR 7.05[1.44-34.45]; P=0.016). NAC patients (criterion 2) had a higher proportion of subjects with incident chronic kidney disease and eye disorders, and NAC patients (criterion 3) had a higher proportion of incident chronic kidney disease, nephrolithiasis and dyslipidemia than AC patients.

**Conclusion:**

The present study shows a strikingly high prevalence of NAC patients in the clinical practice of Spanish endocrinologists. Results suggest that NAC disease might be associated with some prevalent and incident comorbidities.

## Introduction

Hypoparathyroidism is a rare endocrine disease characterized by inappropriately low or insufficient circulating parathyroid hormone (PTH) levels resulting in hypocalcemia and other alterations of mineral metabolism (1, 2). The most common etiology of chronic hypoparathyroidism is inadvertent injury to the parathyroid glands in cervical surgery (postsurgical hypoparathyroidism). These patients have an increased risk of several comorbidities, including increased risk of renal stones and renal failure. Recently, an increased risk of cardiovascular disease has been reported to be associated with chronic hypoparathyroidism (3–6).

Clinical guidelines recommend a periodic evaluation of the clinical and biochemical situation of patients with chronic hypoparathyroidism, as well as an assessment for the presence of comorbidities. The goals of treatment include, on the one hand, clinical well-being with the absence of symptoms of hypocalcemia and an adequate quality of life, and, on the other hand, achieving normal levels of serum adjusted calcium (Ca) and phosphorus (P), a CaxP product below 55 mg^2^/dl^2^, and avoiding hypercalciuria and deterioration of renal function. Usually, patients are managed with conventional therapy of orally administered calcium and active vitamin D (1, 2); however, some remain in a state of poor disease control (2, 7, 8).

The identification of adequately controlled (AC) and not adequately controlled (NAC) patients has a great interest in clinical practice, since the poor control of the disease is related to complications and even mortality, as has been shown in the elegant study by Underbjerg et al. (9). However, only a few studies have evaluated the proportion of patients with chronic hypoparathyroidism who have inadequate control of the disease considering clinical and analytical data (10–12) with results that are difficult to compare, since they depend on the employed criteria. In a recent multicenter and nation-wide study, we analyzed the incidence and risk factors for several comorbidities in a cohort of 337 patients with chronic postsurgical hypoparathyroidism lasting ≥3 years (5). In the present report we aimed to assess the proportion of NAC patients in this national cohort of patients using different disease control criteria, as well as their possible relationships with complications and comorbidities.

## Methods

### Subjects

This study analyzes the clinical and biochemical situation at the end of follow-up in a previously described cohort of patients with chronic hypoparathyroidism after thyroidectomy (5). Briefly, patients included were over 18 years of age at the time of thyroidectomy and had a follow-up period in the same hospital ≥3 years, with a visit between January 1, 2022, and September 15, 2023.

### Study design

This is a multicenter, retrospective cohort study, with data from routine clinical practice, carried out under the auspices of the Thyroid Task Force of the Spanish Society of Endocrinology and Nutrition (SEEN). Twenty investigators from 16 hospital centers agreed to participate in the study. A review of the medical records of all patients who met the inclusion criteria was performed. Each investigator collected information on clinical and demographic data, initial surgery, pathological details, prevalent and incident comorbidities, as well as the clinical situation and biochemical profile at the last visit.

For the purposes of the present study, each investigator evaluated the adequacy or inadequacy of the treatment of hypoparathyroidism in their patients. For this, we used the matrix reported by Iqbal et al. (13) modified and simplified. This matrix uses two axes, one that assesses biochemical values ​​and another that assesses the patient’s well-being. Given that the variables used are considered dichotomous (normal or abnormal biochemical values; the patient is well or not well), four groups can be defined based on the normality or abnormality of the biochemical tests and the patient’s well-being: group 1, patients with normal biochemistry who feel well; group 2, patients with abnormal biochemistry who feel well; group 3, patients with normal biochemistry who feel unwell; and group 4, patients with abnormal biochemistry who feel unwell.

In our study, patient well-being was based on the intensity of symptoms related to hypoparathyroidism, complications of chronic hypoparathyroidism and also other comorbidities or concomitant diseases. Clinicians were required to make a holistic assessment of the patient’s health status. If the clinician believed that any of these symptoms substantially affected the patient’s quality of life, he classified the patient as unwell.

Given that this is a retrospective study, and that not all patients had all the biochemical data available at last visit, three criteria with different levels of demand were used for biochemical classification of patients into normal or abnormal: criterion 1, serum Ca concentrations between 8.0 and 10.5 mg/dl, serum P concentrations between 2.5 and 4.5 mg/dl, and CaxP product <55 mg^2^/dl^2^; criterion 2: normal Ca and P according to the previous criteria, CaxP product <55 mg^2^/dl^2^, and estimated glomerular filtration rate (eGFR) ≥60 ml/min/1.73 m^2^; and criterion 3: the same as in criterion 2 together with a normal 24-hour urinary calcium excretion (<250 mg/24h in females and <300 mg/24h in males).

A patient was considered to be AC when he or she was in group 1 of the matrix. Patients in groups 2, 3 and 4 were considered NAC. Given that in this study three biochemical criteria with different degrees of demand were used, the proportions of NAC patients with lax (criterion 1), intermediate (criterion 2) and demanding (criterion 3) criteria were quantified.

All patient’s data were obtained under the standard medical care conditions. Analytical determinations were performed using routine techniques in the biochemistry laboratories of the participating centers. The Ca values ​​considered in this study refer to serum calcium corrected by albumin or total proteins. The eGFR was calculated by the usual method in each of the participating hospitals (CDK-EPI equation in 60% of the subjects, and MDRD 4-variable equation in 40%). The patient’s confidential information was protected according to national law, and the study received favorable report from the ethics committee of the Hospital Universitario Puerta de Hierro Majadahonda (PI 253/22). Furthermore, before its dissemination, the boards of directors of SEEN approved the study.

### Statistical analysis

For quantitative variables, results are expressed as median (interquartile range, IQR). Categorical variables are described as absolute values, ratios, or percentages. For proportion comparisons, the chi-square test or Fisher’s exact test was used. For comparisons of means between two groups of subjects the Mann–Whitney U-test was employed. Several univariate and multivariable logistic regression models were used to explore the factors determining the inadequacy of disease control. Clinical, demographic, and therapeutic data were included as explanatory variables in these models. Multivariable model 1 was adjusted for prevalent comorbidities at the time of diagnosis of hypoparathyroidism; model 2 was adjusted for the same covariates, and demographic data. All used tests were two-sided and differences were considered significant when P < 0.05.

## Results

### Studied patients

The studied cohort consisted of 337 patients (84.3% women, median age 45[36-56] years, median time of follow-up 8.9[6.0-13.0] years). The cause of thyroidectomy was thyroid cancer in 255 patients (66.8%) and benign thyroid disease in the rest. All patients were treated by conventional therapy of chronic hypoparathyroidism with orally administered calcium (n=296; median dose 1.25[1-2] g/day) and calcitriol (n=332; 0.50[0.25-0.50] μg/day).

**Table 1** shows the biochemical data at the end of follow-up with an indication of the number of patients in which each parameter was available. As shown, 83.6% (249 out of 298) and 64.9% (211 out of 325) of patients had normal serum Ca and P levels, respectively. Two hundred and seventy one out of 306 patients (88.6%) showed an eGFR greater than 60 ml/min/1.73m^2^. Normal calciuria was obtained in 85 of 123 women (69.1%) and in 27 of 29 men (93.1%) with this parameter available.

**Table 1:** Characteristics of patients with chronic hypoparathyroidism and assessment of the biochemical and clinical normality criteria at the end of the follow-up period.

	Units or criteria	N	Median	IQR	Number	Percentage
*Clinical characteristics*						
**Gender**	Female	337			284	84.3
**Age**	Year	337	45.0	36.0-55.9		
**Time of follow-up**	Year	337	8.9	6.0-13.0		
**Histopathology**	Thyroid cancer	337			225	66.8
*Biochemical values*						
**Ca**	mg/dl	298	8.6	8.2-9.0		
**Normal Ca**	8.0-10.5 mg/dl	298			249	83.6
**P**	mg/dl	325	4.3	3.8-4.8		
**Normal P**	3.5-4.5 mg/dl	325			211	64.9
**Ca x P product**	mg^2^/dl^2^	286	36.6	32.4-40.8		
**Normal Ca x P product**	<55 mg^2^/dl^2^	286			284	99.3
**eGFR**	ml/min/1.73m^2^	306	84.0	71.8-90.0		
**Normal eGRF**	≥60 ml/min/1.73m^2^	306			271	88.6
**Calciuria (males)**	mg/24 h	29	224.0	138.0-271.5		
**Calciuria (females)**	mg/24 h	123	200.0	134.0-268.8		
**Normal calciuria (males)**	<300 mg/24 h	29			27	93.1
**Normal calciuria (females)**	<250 mg/24 h	123			85	69.1
**Normal calciuria (all)**		152			112	73.7
*Assessment at last visit*						
Normal biochemical levels						
**Criterion 1**		286			165	57.7
**Criterion 2**		257			139	54.1
**Criterion 3**		112			45	40.2
**Normal clinical assessment**		334			289	85.0

N, number of patients in which the parameter is available; IQR, interquartile range; Ca, corrected serum calcium; P, serum phosphorus; Ca x P, serum calcium–phosphate product; eGFR, estimated glomerular filtration rate.

Biochemical criteria: normal criterion 1 applies to patients with normal serum calcium and phosphorus levels and normal Ca x P product; normal criterion 2 implies normality of criterion 1 plus normal eGFR; normal criterion 3 implies the normality of criterion 2 plus normal calciuria.

As expected, normality for the biochemical criteria was obtained in a proportion of patients that decreased as the requirement of the criterion increased: 165 of 286 patients (57.7%) for criterion 1, 139 of 257 (54.1%) for criterion 2, and 45 of 112 (40.2%) for criterion 3. The doctors’ assessment of the general clinical situation at the end of follow-up showed a normal result in 289 of 334 (85.0%) patients.

### AC and NAC patients

**Figure 1:**
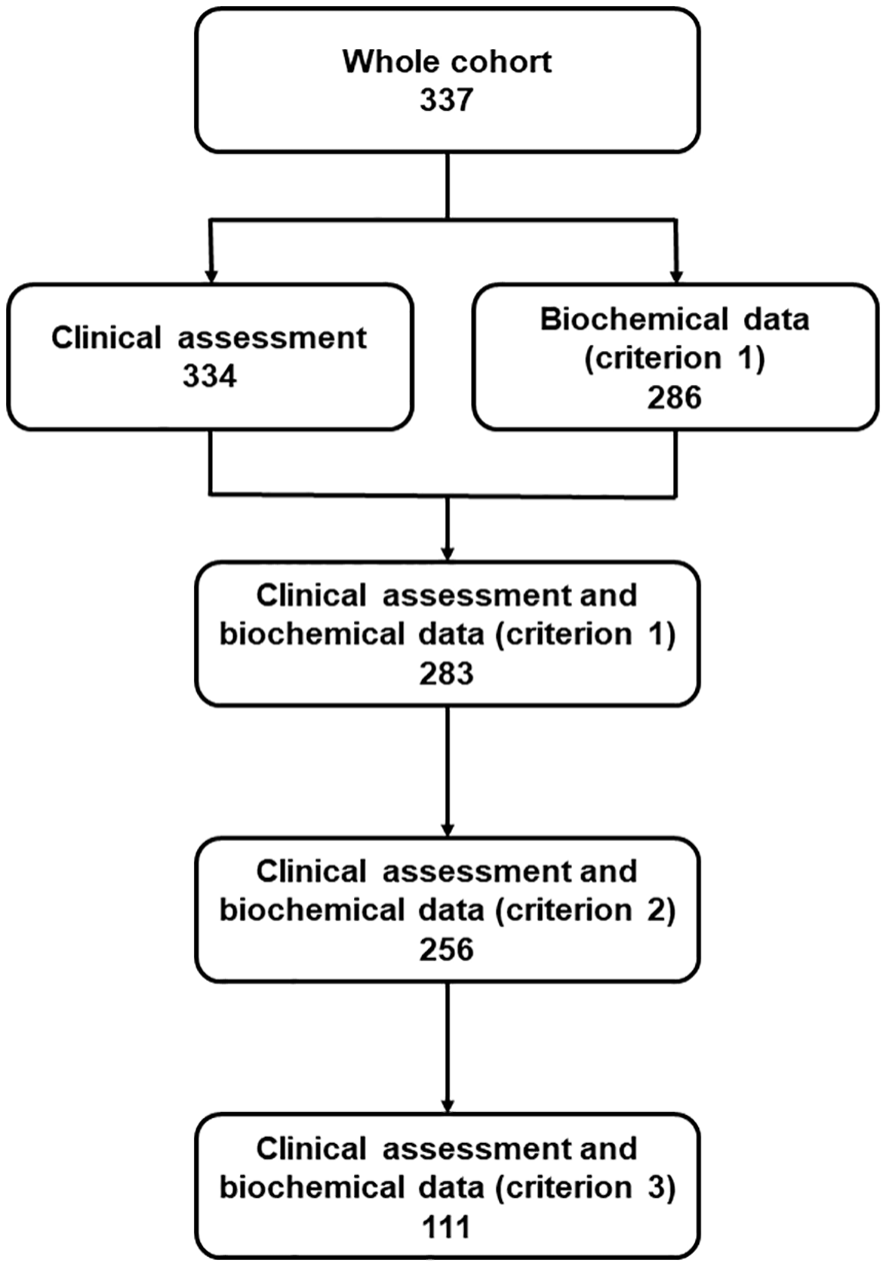
Flow diagram indicating the sample sizes of each group of patients with availability of clinical and biochemical data according to the three control criteria used in the study.

**Figure 2:**
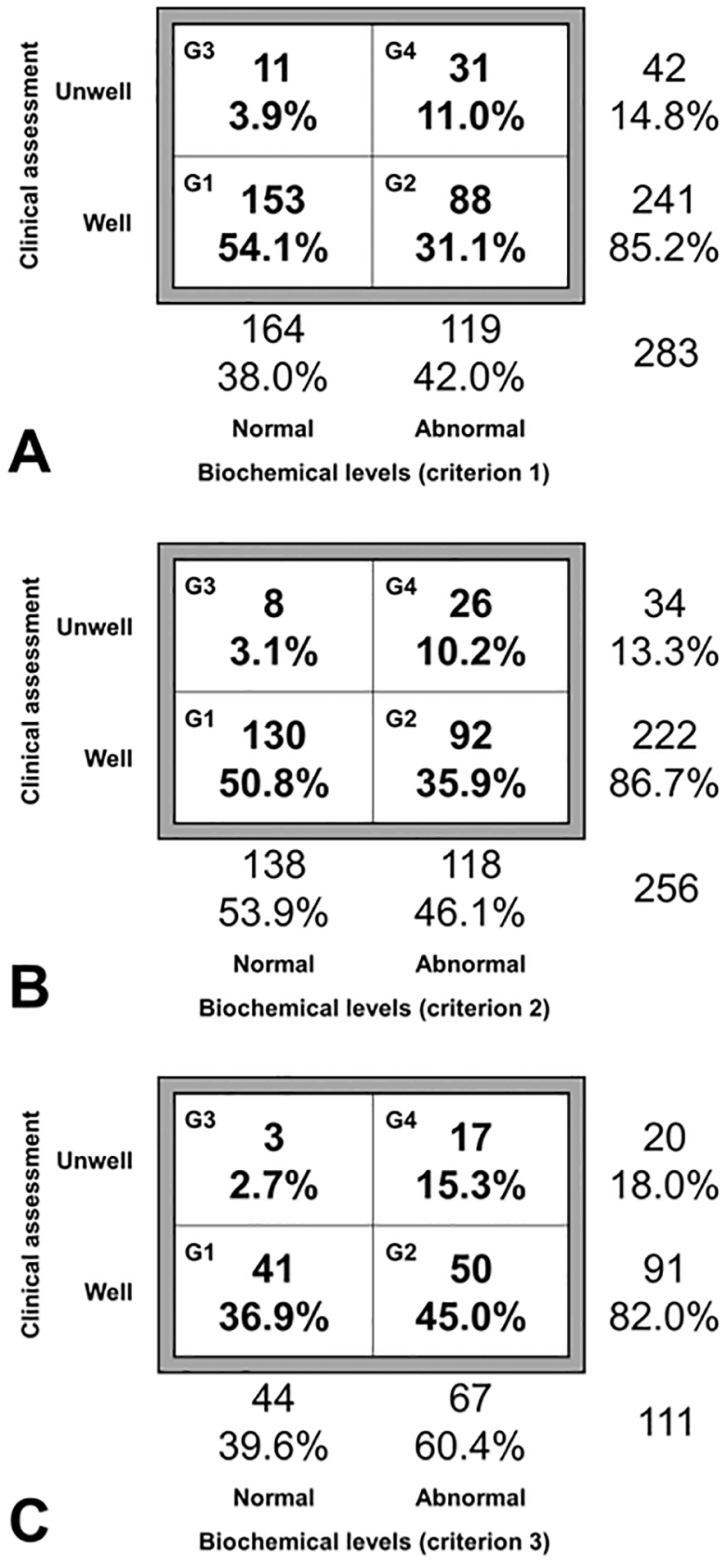
Patient classification matrices according to biochemical levels and the clinical assessment at the end of follow-up. The data are the absolute number and percentage of patients in each group or subgroup. Each panel represents one of the biochemical criteria: **(A)**, criterion 1; **(B)**, criterion 2; **(C)**, criterion 3. G1, group 1 (normal biochemical levels/well); G2, group 2 (abnormal biochemical levels/well); G3, group 3 (normal biochemical levels/unwell); G4, group 4 (abnormal biochemical levels/unwell).

The number of patients with available data that we used to draw the three matrices and calculate the proportion of NAC patients according to the three criteria is shown in **Figure 1**. The matrices displaying the disease control assessment, including the absolute values ​​and percentages of patients in the four groups, are shown in **Figure 2**. As shown, the proportion of AC patients (group 1) was clearly higher in matrix 1 (153/283; 54.1%) compared to matrix 3 (41/111; 36.9%). The opposite was seen in the case of group 4, that is, patients with poor biochemical and clinical control (31/283; 11.0% in matrix 1 vs. 17/111; 15.3% in matrix 3).

### Relationship of control of hypoparathyroidism with prevalent comorbidities

To assess the relationship between disease control and demographic and clinical data at the time of diagnosis, the values found at diagnosis in AC and NAC patients were compared based on the three control criteria (**Table 2**). No significant relationships were found, with any of the three employed criteria, between disease control at the end of follow-up and gender, age, time of evolution or histology of the surgical specimen. Furthermore, there were no differences between AC and NAC patients when criterion 1 was applied in any of the studied prevalent comorbidities. When criterion 2 was employed, NAC patients had a significant higher prevalence at the time of diagnosis of chronic kidney disease, hypertension, dyslipidemia and cardiovascular disease. However, with the strictest criterion, the difference was only significant for the prevalence of dyslipidemia.

**Table 2:** Relationship between disease control at the end of follow-up and demographic and clinical characteristics, and prevalent diseases at the time of diagnosis of hypoparathyroidism.

	Control of hypoparathyroidism at the end of follow-up according to three criteria								
	Criterion 1			Criterion 2			Criterion 3		
	NAC	AC	P	NAC	AC	P	NAC	AC	P
**Number of patients**	130	153		126	130		70	41	
**Gender, female**	110 (84.6)	127	0.749	106 (84.1)	109 (83.8)	1.0	58 (82.9)	29	0.156
**Age, yr**	44 (34-54)	46.0	0.476	48 (37-57)	43	0.057	48 (37-56)	43 (35-51)	0.181
**Time of follow-up, yr**	9 (6-14)	9 (6-13)	0.732	8 (5-12)	9 (7-13)	0.233	9 (6-14)	9 (8-13)	0.893
**Histology, cancer**	92 (70.8)	99 (67.4)	0.309	87 (69.0)	87 (66.9)	0.789	45 (64.3)	29 (70.7)	0.537
**Chronic kidney disease**	3 (2.3)	4 (2.6)	1.0	7 (5.6)	0	**0.006**	1 (1.4)	0	1.0
**Nephrolithiasis**	3 (2.3)	1 (0.7)	0.337	4 (3.2)	0	0.057	1 (1.4)	0	1.0
**Hypertension**	22 (16.9)	26 (17.0)	1.0	30 (23.8)	17 (13.1)	**0.035**	14 (20.0)	4 (9.8)	0.190
**Dyslipidemia**	31 (23.8)	27 (17.6)	0.237	35 (27.1)	19 (14.6)	**0.014**	20 (28.6)	2 (4.9)	**0.003**
**Diabetes**	6 (4.6)	7 (4.6)	1.0	9 (7.1)	3 (2.3)	0.081	3 (4.3)	0	0.295
**Cardiovascular disease**	6 (4.6)	3 (2.0)	0.310	8 (6.3)	1 (0.8)	**0.018**	1 (1.4)	0	1.0
**CNS disease**	3 (2.3)	2 (1.3)	0.664	4 (3.2)	1 (0.8)	0.208	2 (2.9)	0	0.530
**Mental health disorders**	20 (15.4	19 (12.4)	0.493	24 (19.0)	15 (11.5)	0.117	10 (14.3)	5 (12.2)	1.0
**Eye disorders**	4 (3.1)	5 (3.3)	1.0	5 (4.0)	3 (2.3)	0.495	2 (2.9)	2 (4.5)	0.625
**BMD alterations**	4 (3.1)	3 (2.0)	0.707	4 (3.2)	3 (2.3)	0.719	2 (2.9)	1 (2.4)	1.0
**Fracture**	1 (0.8)	2 (1.3)	1.0	1 (0.8)	2 (1.5)	1.0	1 (1.4)	1 (2.4)	1.0
**Cancer**	7 (5.4)	10 (6.5)	0.804	8 (6.3)	9 (6.9)	1.0	3 (4.3)	3 (7.3)	0.668

NAC, not adequately controlled patients; AC, adequately controlled patients; CNS, central nervous system; BMD, bone mineral density. Values highlighted in bold indicate statistically significant differences.

Several models of logistic regression analysis were performed to study the dependence of the variable inadequate disease control as a function of several independent variables (**Supplementary Material**; **Supplementary Tables S1-S3**). When criteria 1 and 2 were used, none of the studied variables were significant in multivariable models. However, when we used criterion 3, patients who had dyslipidemia at the time of diagnosis presented a significantly higher risk of inadequate control of hypoparathyroidism in both univariable (OR 7.80[1.72-35.40]; P=0.008) and multivariable models (OR 7.05[1.44-34.45]; P=0.016).

### Incident comorbidities in AC and NAC patients

**Table 3** shows the proportion of patients who presented incident comorbidities throughout follow-up in subjects classified according to their degree of control and according to the criteria for evaluating disease control. There were no differences in the incidence of comorbidities when criterion 1 was applied. However, with criterion 2, we observed that NAC patients had a higher proportion of subjects with incident chronic kidney disease and eye disorders than those found in AC patients. Lastly, NAC patients with criterion 3 exhibited a higher proportion of incident chronic kidney disease, nephrolithiasis and dyslipidemia than AC patients.

**Table 3:** Incident comorbidities in patients classified according to the degree of adequacy of hypoparathyroidism control at the last visit.

	Control of hypoparathyroidism at the end of follow-up according to three criteria								
	Criterion 1			Criterion 2			Criterion 3		
	NAC	AC	P	NAC	AC	P	NAC	AC	P
	130	153		126	130		70	41	
**Chronic kidney disease**	11 (8.5)	11 (7.2)	0.824	12 (16.7)	1 (0.8)	**<0.001**	15 (21.4)	0	**0.001**
**Nephrolithiasis**	10 (7.7)	8 (5.2)	0.467	11 (8.7)	6 (4.6)	0.216	13 (18.6)	1 (2.4)	**0.016**
**Hypertension**	16 (12.3)	26 (17.0)	0.316	20 (15.9)	18 (13.8)	0.726	13 (18.6)	3 (7.3)	0.161
**Dyslipidemia**	23 (17.7)	26 (17.0)	0.876	25 (19.8)	17 (13.1)	0.177	20 (28.6)	3 (7.3)	**0.008**
**Diabetes**	7 (5.4)	13 (8.5)	0.358	7 (5.6)	11 (8.5)	0.465	7 (10.0)	1 (2.4)	0.254
**Cardiovascular disease**	8 (6.2)	16 (10.5)	0.208	11 (8.7)	11 (8.5)	1.0	7 (10.0)	1 (2.4)	0.254
**CNS disease**	1 (0.8)	0	0.459	1 (0.8)	0	0.492	1 (1.4)	0	1.0
**Mental health disorders**	19 (14.6)	21 (13.7)	0.865	17 (13.5)	19 (14.6)	0.858	15 (21.4)	6 (14.6)	0.457
**Eye disorders**	9 (6.9)	3 (2.0)	0.072	10 (7.9)	2 (1.5)	**0.018**	4 (5.7)	1 (2.4)	0.650
**BMD alterations**	9 (6.9)	10 (6.5)	1.0	10 (7.9)	9 (6.9)	0.815	4 (5.7)	2 (4.9)	1.0
**Fracture**	1 (0.8)	0	0.459	1 (0.8)	0	0.492	0	0	—
**Cancer**	7 (5.4)	11 (7.2)	0.629	7 (5.6)	8 (6.2)	1.0	7 (10.0)	1 (2.4)	0.254

NAC, not adequately controlled patients; AC, adequately controlled patients; CNS, central nervous system; BMD, bone mineral density. Values ​​highlighted in bold indicate statistically significant differences.

## Discussion

Results of this study provide two clinically relevant novelties in the field of care for patients with hypoparathyroidism. Firstly, our data reveal a strikingly high proportion of patients with poor control of the disease. In this cohort, NAC patients were 45.9%, 49.2% and 63.1% with criteria 1, 2 and 3, respectively. These data highlight that, even with the most lenient criterion, almost half of the patients with chronic hypoparathyroidism in the clinical practice of Spanish endocrinologists are poorly controlled. Secondly, our data also suggest that patients who, at the time of diagnosis of hypoparathyroidism, present with chronic kidney disease, hypertension, dyslipidemia, and cardiovascular disease have a greater tendency toward poor disease control with the intermediate criterion. However, only dyslipidemia is associated with poor disease control when the strictest biochemical criterion is applied (criterion 3). The interpretation of these results requires considering the distinct criteria used in our analysis. The difference between criterion 1 and criterion 2 is the requirement of an eGFR ≥60 ml/min/1.73m^2^ in the latter. This may explain the relationships found in NAC patients with criterion 2 with both prevalent and incident chronic kidney disease.

The matrix used in this study is a variant of that used by Iqbal et al. (13) in a European Delphi panel and by other authors in Spain (14), The Netherlands and Belgium (15). The biochemical criteria of our study have been very similar to those originally described by Iqbal et al., although there are some differences. These authors considered the presence of hypocalcemia on at least 3-4 annual measurements as abnormal biochemical criterion. In our study we have used only the Ca value from the last follow-up visit. Our data indicate that there are few differences in the assessment of biochemical control using criteria 1 and 2, but a huge difference when criterion 3 is applied. Criterion 3 of our study included the measurement of calciuria; therefore, it could only be applied in 33% of the cohort, due to the absence of this parameter in a large proportion of patients. These results should make us recognize two aspects: first, the importance of measuring calciuria in all patients and, second, the convenience of the inclusion of calciuria in the biochemical criteria for assessing the adequacy of treatment for hypoparathyroidism.

The second axis of the matrix refers to the clinical assessment of the patient. Regarding the wellness of the patients, our study uses clinical assessment at the discretion of the treating doctor, in a way similar to that of previous studies (13, 14), and taking into account the current situation and the whole history of the patient. This form of clinical assessment has the disadvantage that the clinician’s opinion is conditioned by the data available in the clinical record, as well as by the exhaustiveness of the questioning of the symptoms, which may vary between the different physicians participating in the study. However, all participants were endocrinologists who are experts in the follow-up of patients with chronic hypoparathyroidism and they carried out a holistic assessment of the patient’s well-being, which included not only the typical symptoms of hypocalcemia, but also those derived from complications of hypoparathyroidism and the patient’s comorbidities that may affect their health status. The subjective component in patients’ assessment of symptoms may be a limiting factor in this study. However, there is a growing trend to use patient-reported outcome measures in clinical practice as a way to optimize person-centered and value-based healthcare (16).

It is important to note that few studies have addressed the epidemiology and natural history of chronic hypoparathyroidism (3, 17–21); but more striking is that information on the adequacy of disease control is even scarcer (10–12). Our study attempts to shed some light on this knowledge gap. The high prevalence of poor control in this study contrasts with the finding of another previous study by our group (12), in which 15% of a cohort of 260 patients with chronic hypoparathyroidism were identified as NAC. The differences may be explained by patient selection criteria and definition of poor control. In our first study, only a disease progression time of at least one year was required, and a patient was considered to be NAC if he or she had serum Ca <8 mg/dl or hypercalciuria defined as >300 mg/24 h in females or >400 mg/24h in males (12). In summary, the biochemical control criteria were less strict than in the present study.

The clinical evaluation of patients with hypoparathyroidism is not free of difficulties nor are there criteria clearly established by clinical guidelines (22). It is likely that, in our study, as in others (11), the inadequacy of symptomatic control of the disease is undervalued. Certainly, in our study there was no common system for collecting information due to the retrospective nature of the research. It is likely that if a defined questionnaire of clinical symptoms had been used in routine patient check-ups, a higher proportion of patients with poor clinical control would have been found. In fact, a survey performed in the US reported that 72% of patients had experienced more than ten hypoparathyroidism-related symptoms, and 45% reported that their symptoms significantly interfered in their life (23).

This poor symptom control has an impact on quality of life, as has been documented in different studies (21, 23–25). A recent survey showed that 85% and 67% of NAC patients had neuromuscular and neurological symptoms, respectively (15). A study that used several instruments to measure quality of life demonstrated that the patient’s self-rated symptom severity was moderate or severe in 53% and 12% of the patients, respectively. The magnitude of symptoms severity reported by NAC patients was inversely related to quality of life and health status scores (26).

Our study shows that alterations in mineral metabolism are the most common cause of inadequate control of patients with hypoparathyroidism. In accordance with our results, data from an Italian registry, including 509 patients with hypoparathyroidism, showed that 43.8% had low serum Ca levels and that hypercalciuria was present in 39% of patients (10). Our cohort showed somewhat different values: abnormal serum Ca in 16.4% of patients and abnormal calciuria in 6.9% of men and 30.9% of women. Furthermore, our study also included the assessment of serum P (abnormal in 35.1% of patients) and eGFR (abnormal in 11.4%). This explains that, combining the biochemical data with the three criteria and the clinical data, our percentages of inadequacy reached values ​​between 45.9% and 63.1%, more similar to those reported by Marcucci et al. (10). Some differences between the Italian registry and our study should be mentioned. In the Italian study, only 67.6% of the cases were postsurgical. Furthermore, the median age of the patients in our study (45 years) was clearly higher than that of the patients in the Italian study (24 years), which included 9.4% of subjects aged less than 18 years. A limiting factor of our criterion 3 is that it could only be evaluated in 111 subjects (33% of the total cohort of 337). This is because, as in other studies (10), calciuria was only available in a fraction of patients.

The retrospective multi-country chart review by Chen et al. (11) analyzed a cohort of 614 patients with hypoparathyroidism. Similar to our study, the majority (61.6%) were women and had postsurgical hypoparathyroidism (74.4%). However, the mean duration of disease was shorter (46 months) than that of our cohort. In the study by Chen et al. (11) the percentage of NAC patients was 28%, using serum Ca, serum P and calciuria criteria. Again, this value of inadequacy is clearly lower than that found in our study with the most demanding criterion (criterion 3), which requires normality not only of calciuria but also of eGFR. On the other hand, clinicians’ estimate of inappropriateness was only 11% in Chen’s study. In our survey, the assessment at the physician’s discretion was only applied to the patient’s well-being. According to this, 15% of the patients felt unwell, a value similar to that reported by Chen. In both cases, it can be seen that the objective assessment using biochemical data offers values ​​of poor control that are clearly higher than those obtained through subjective assessment or the doctor’s opinion.

The analytical assessment of patients with hypoparathyroidism is important not only for treatment adjustments, but also for its relationship with different comorbidities. The study by Underbjerg et al. (9) showed that patients with time-weighted low serum Ca had a higher risk of cardiovascular disease and patients with time-weighted high P had a higher risk of mortality and infection.

Similar to our previous study (12), in the current study we did not find significant differences between AC and NAC patients in gender, age, time of follow-up and histopathological features. The relationships found between NAC patients with criterion 2 and the presence of hypertension, dyslipidemia and prevalent cardiovascular disease are interesting from a clinical point of view, since they can serve as an alert for closer monitoring of patients with chronic hypoparathyroidism who present these comorbidities. However, in the multivariate analysis, no significant associations were found with criterion 2, but we were able to demonstrate an influence of dyslipidemia on poor disease control with criterion 3. There are experimental data suggesting that PTH may have non-classical actions in different locations such as adipose tissue, vascular wall and myocardium (27). Observational studies have found an association between dyslipidemia and primary hyperparathyroidism (28, 29). Recently, the association with dyslipidemia has also been reported in patients with hypoparathyroidism (5, 15). Therefore, it seems plausible that alterations in PTH secretion, together with the changes in vitamin D metabolites that accompany it, could have metabolic effects on lipid levels (30). Nevertheless, our finding on the relationship between dyslipidemia and NAC patients is not easy to explain from a mechanistic point of view. Interpretation of our results from a global point of view would suggest that patients with a higher initial level of morbidity present a certain tendency toward poor control of hypoparathyroidism.

Our data also suggest that there is an association between poor control of hypoparathyroidism and some incident comorbidities, an aspect that may also be of interest for clinical follow-up. NAC patients have a higher incidence of eye disorders than AC patients (criterion 2) and also a higher incidence of chronic kidney disease, nephrolithiasis and dyslipidemia (criterion 3). Again, part of these relationships are explained by the requirement of normality of the eGFR to classify a patient as AC with criteria 2 and 3. Concerning eye disorders, it is known that hypoparathyroidism is associated with an increased risk of cataract ad papilledema, especially in non-surgical patients and those with a longer duration of the disease (22, 31, 32). Compared to subjects without PTH abnormalities, patients with hypoparathyroidism present this complication at younger ages and show predominantly cortical involvement (33). Although the mechanism of cataract formation is unclear, ocular complications have been related to chronic hypocalcemia (34). Therefore, it seems reasonable to assume that patients with worse biochemical control of the disease will have a higher prevalence of eye disorders.

Evaluated from an overall point of view, these data suggest that there is also a relationship between the inadequacy of the control of hypoparathyroidism and the disease burden that is acquired throughout the natural history of the disease. Furthermore, this result is consistent with our previous data (12) which showed that hospitalization rate during follow-up was significantly higher in the group of NAC patients (35.9%) compared with AC patients (10.9%). In accordance with this, Chen et al. (11) also showed that NAC patients had not only a greater number of hospitalizations than AC patients, but also a greater number of outpatient visits and emergency room visits.

Our results may have some implications for clinical practice. Although in the last years many studies on the prevalence of permanent hypoparathyroidism after cervical surgery (35–37), and recent clinical guidelines and consensus statements on the management and treatment of this hormonal deficiency (22, 38) have been published, few studies have attempted to define and quantify the proportion of patients who do not achieve adequate control of the disease (10, 12). Our study shows that a large percentage of patients are not well controlled, even with the most permissive control criteria (criterion 1), from which it follows that conventional treatment with orally administered calcium and active vitamin D (1, 2) is clearly insufficient to achieve clinical well-being and normalization of patient analytical data. Assessment of the adequacy of hypoparathyroidism control is a challenge for clinicians involved in the care of these patients and should be performed as rigorously as possible. The results obtained in this real-life cohort allow us to highlight the importance of measuring calciuria in the follow-up of patients. The absence of this measurement in a large proportion of patients in this study may serve as a wake-up call to clinicians to incorporate the quantification of urinary calcium excretion in their routine follow-up of patients with chronic hypoparathyroidism. Furthermore, poor control of the disease negatively affects the personal relationships, work productivity, and the ability to carry out daily activities not only of the patients, but also of their caregivers (26).

Our study is an analysis of real-life clinical practice, and its data reflect the clinical routine of endocrinology specialists with expertise in the treatment of postsurgical hypoparathyroidism. The relatively high sample size of the cohort, as well as the multicenter and nationwide design, and the long duration of the disease, are strengths of our study. However, the variable availability of patients’ analytical data at the last visit is a limitation of our study that we must recognize. This has prevented us from analyzing the proportion of NAC patients in the entire cohort. We specifically have to recognize that the challenge in biochemical management of hypoparathyroidism really lies in criteria 3. However, our cohort presents a large proportion of patients in whom calciuria was not available, which limits the assessment using criterion 3 to only 111 patients (33%). On the other hand, the assessment of some comorbidities in our study presents some limitations. In particular, we acknowledge that the use of anamnesis or records of fractures with clinical manifestations are not the most appropriate procedures to investigate the impact of hypoparathyroidism on the skeleton. Recently, it has been demonstrated that vertebral fracture assessment (VFA) is a safe and reliable methodology for vertebral fracture diagnosis and allows the detection of fractures despite normal bone mineral density values ​​in postmenopausal women with postsurgical hypoparathyroidism (39). Other limitations include the retrospective nature of the study and the difference in patient monitoring and treatment protocols at different hospitals. We also acknowledge that the assessment of patient well-being is limited by the absence of a defined clinical protocol for assessing patient symptoms or validated quality of life questionnaires. Our study was limited to the setting of specialized medical care in Spain; therefore, results cannot be extrapolated to other countries or settings.

In summary, the present study shows a high prevalence of NAC patients in the clinical practice of Spanish endocrinologists with expertise in hypoparathyroidism. The main cause of NAC disease is the lack of normalization of the biochemical parameters of mineral metabolism. Our data also suggest that inadequacy is associated with prevalent and incident comorbidities, although further studies are necessary to confirm these results and define the risk factors and determinants of inadequacy in the control of chronic hypoparathyroidism.

## Data Availability

The raw data supporting the conclusions of this article will be made available by the authors, without undue reservation.
