# Supplementary material for: Prevalence of chronic postsurgical hypoparathyroidism not adequately controlled: an analysis of a nationwide cohort of 337 patients
Source: Front Endocrinol (Lausanne). 2024 Sep 25;15:1464515. doi: 10.3389/fendo.2024.1464515 (PMC11461294; doi:10.3389/fendo.2024.1464515)
Supplement: Supplementary file 1 [file Table1.docx]

**Prevalence of chronic postsurgical hypoparathyroidism not adequately controlled: an analysis of a nationwide cohort of 337 patients**

**SUPPLEMENTARY MATERIAL**

**Table S1**. Results of univariable and multivariable logistic regression models to study the influence of several covariates at the time of diagnosis as potential predictors of treatment inadequacy at the end of follow-up (criterion 1)

|  | **Univariable** | | | **Model 1** | | | **Model 2** | | |
| --- | --- | --- | --- | --- | --- | --- | --- | --- | --- |
|  | **OR** | **95% CI** | **P** | **OR** | **95% CI** | **P** | **OR** | **95% CI** | **P** |
| **Chronic kidney disease** | 0.88 | 0.19-4.01 | 0.869 | 0.68 | 0.12-3.87 | 0.661 | 0.73 | 0.12-4.30 | 0.728 |
| **Nephrolithiasis** | 3.59 | 0.37-34.94 | 0.271 | 3.32 | 0.34-32.91 | 0.305 | 3.52 | 0.35-35.24 | 0.284 |
| **Hypertension** | 0.99 | 0.53-1.86 | 0.897 | 0.79 | 0.38-1.61 | 0.508 | 0.98 | 0.44-2.16 | 0.954 |
| **Dyslipidemia** | 1.46 | 0.82-2.61 | 0.199 | 1.43 | 0.73-2.78 | 0.294 | 1.65 | 0.82-3.33 | 0.160 |
| **Diabetes** | 1.01 | 0.33-3.08 | 0.987 | 0.92 | 0.25-3.41 | 0.897 | 0.85 | 0.23-3.19 | 0.850 |
| **Cardiovascular disease** | 2.42 | 0.59-9.87 | 0.218 | 2.49 | 0.54-11.42 | 0.240 | 2.49 | 0.54-11.42 | 0.239 |
| **Mental health disorders** | 1.28 | 0.65-2.52 | 0.471 | 1.18 | 0.58-2.37 | 0.648 | 1.23 | 0.60-2.50 | 0.574 |
| **Gender, female** | 1.13 | 0.60-2.13 | 0.715 |  |  |  | 1.17 | 0.60-2.26 | 0.648 |
| **Age, yr** | 0.99 | 0.98-1.01 | 0.527 |  |  |  | 0.99 | 0.97-1.01 | 0.238 |
| **Time of follow-up, yr** | 1.01 | 0.91-1.05 | 0.777 |  |  |  | 1.01 | 0.97-1.05 | 0.707 |

Abbreviations: OR odds ratio, CI confidence interval.

Model 1: prevalent comorbidities including chronic kidney disease, nephrolithiasis, hypertension, dyslipidemia, diabetes, cardiovascular disease and mental health disorders; model 2: in addition to the above, demographic features (gender, age) and time of follow-up.

**Table S2**. Results of univariable and multivariable logistic regression models to study the influence of several covariates at the time of diagnosis as potential predictors of treatment inadequacy at the end of follow-up (criterion 2)

|  | **Univariable** | | | **Model 1** | | | **Model 2** | | |
| --- | --- | --- | --- | --- | --- | --- | --- | --- | --- |
|  | **OR** | **95% CI** | **P** | **OR** | **95% CI** | **P** | **OR** | **95% CI** | **P** |
| **Hypertension** | 2.08 | 1.08-4.00 | **0.029** | 1.37 | 0.66-2.83 | 0.400 | 1.28 | 0.57-2.91 | 0.554 |
| **Dyslipidemia** | 2.25 | 1.21-4.19 | **0.011** | 1.52 | 0.75-3.09 | 0.248 | 1.47 | 0.70-3.08 | 0.314 |
| **Diabetes** | 3.26 | 0.86-12.32 | 0.082 | 1.79 | 0.40-7.92 | 0.443 | 1.84 | 0.41-8.17 | 0.425 |
| **Cardiovascular disease** | 8.75 | 1.08-70.98 | **0.042** | 5.16 | 0.59-44.85 | 0.137 | 5.38 | 0.61-41.48 | 0.130 |
| **Mental health disorders** | 1.80 | 0.90-3.63 | 0.098 | 1.57 | 0.76-3.24 | 0.226 | 1.54 | 0.74-3.21 | 0.253 |
| **Gender, female** | 1.02 | 0.52-1.99 | 0.851 |  |  |  | 1.07 | 0.53-2.19 | 0.845 |
| **Age, yr** | 1.02 | 1.00-1.04 | 0.051 |  |  |  | 1.01 | 0.98-1.63 | 0.694 |
| **Time of follow-up, yr** | 0.99 | 0.95-1.03 | 0.562 |  |  |  | 1.01 | 0.96-1.05 | 0.828 |

Abbreviations: OR odds ratio, CI confidence interval.

Model 1: prevalent comorbidities including hypertension, dyslipidemia, diabetes, cardiovascular disease and mental health disorders; model 2: in addition to the above, demographic features (gender, age) and time of follow-up. Bold values indicate statistically significant values.

**Table S3**. Results of univariable and multivariable logistic regression models to study the influence of several covariates at the time of diagnosis as potential predictors of treatment inadequacy at the end of follow-up (criterion 3)

|  | **Univariable** | | | **Model 1** | | | **Model 2** | | |
| --- | --- | --- | --- | --- | --- | --- | --- | --- | --- |
|  | **OR** | **95% CI** | **P** | **OR** | **95% CI** | **P** | **OR** | **95% CI** | **P** |
| **Hypertension** | 2.31 | 0.71-7.57 | 0.166 | 1.39 | 0.36-5.34 | 0.634 | 1.50 | 0.35-6.48 | 0.589 |
| **Dyslipidemia** | 7.80 | 1.72-35.40 | **0.008** | 7.13 | 1.52-33.54 | **0.013** | 7.05 | 1.44-34.45 | **0.016** |
| **Mental health disorders** | 1.20 | 0.38-3.79 | 0.756 | 0.98 | 0.28-3.48 | 0.975 | 0.92 | 0.26-3.28 | 0.892 |
| **Gender, female** | 2.00 | 0.80-3.00 | 0.138 |  |  |  | 2.03 | 0.75-5.53 | 0.166 |
| **Age, yr** | 1.02 | 0.99-1.05 | 0.218 |  |  |  | 0.99 | 0.96-1.04 | 0.955 |
| **Time of follow-up, yr** | 1.01 | 0.93-1.10 | 0.840 |  |  |  | 1.04 | 0.94-1.15 | 0.435 |

Abbreviations: OR odds ratio, CI confidence interval.

Model 1: prevalent comorbidities including hypertension, dyslipidemia, and mental health disorders; model 2: in addition to the above, demographic features (gender, age) and time of follow-up. Bold values indicate statistically significant values.
